# A Cross-Sectional Analysis of Oil Pulling on YouTube Shorts

**DOI:** 10.3390/dj13070330

**Published:** 2025-07-21

**Authors:** Jun Yaung, Sun Ha Park, Shahed Al Khalifah

**Affiliations:** 1 School of Dentistry, University of California, Los Angeles, Los Angeles, CA 90024, USA; jun.yaung@ucla.edu (J.Y.); sunhapark19@ucla.edu (S.H.P.); 2 Section of Restorative Dentistry, School of Dentistry, University of California, Los Angeles, Los Angeles, CA 90024, USA

**Keywords:** YouTube Shorts, oil pulling, social media, oral health

## Abstract

**Objective**: This cross-sectional content analysis aimed to investigate how oil pulling is portrayed on YouTube Shorts, focusing on the types of speakers, claims made, and alignment with scientific evidence. The study further explored how the content may influence viewer perception, health behaviors, and the potential spread of misinformation. **Methods**: On 28 January 2025, a systematic search of YouTube Shorts was performed using the term “oil pulling” in incognito mode to reduce algorithmic bias. English language videos with at least 1000 views were included through purposive sampling. A total of 47 Shorts met the inclusion criteria. Data were extracted using a structured coding framework that recorded speaker type (e.g., dentist, hygienist, influencer), engagement metrics, stated benefits, oil type and regimen, the use of disclaimers or citations, and stance toward oil pulling rated on a 5-point Likert scale. Speaker background and nationality were determined through publicly available channel descriptions or linked websites, with user identities anonymized and ethical approval deemed unnecessary due to the use of publicly available content. In total, 47 videos met the inclusion criteria. **Results**: Of the 47 YouTube Shorts that met the inclusion criteria, most were posted by influencers rather than dental professionals. These videos predominantly encouraged oil pulling, often recommending coconut oil for 10–15 min daily and citing benefits such as reduced halitosis and improved gum health. However, a smaller subset advanced more extreme claims, including reversing cavities and remineralizing enamel. Notably, US-licensed dentists and dental hygienists tended to discourage or express skepticism toward oil pulling, assigning lower Likert scores (1 or 2) to influencers and alternative health practitioners (often 4 or 5). **Conclusions**: YouTube Shorts largely promote oil pulling through anecdotal and testimonial-driven content, often diverging from evidence-based dental recommendations. The findings reveal a disconnect between professional dental guidance and popular social media narratives. While some benefits like halitosis reduction may have limited support, exaggerated or misleading claims may result in improper oral hygiene practices. Greater engagement from dental professionals and improved health communication strategies are needed to counteract misinformation and reinforce oil pulling’s role, if any, as an adjunct—not a replacement—for standard oral care. Future studies should explore viewer interpretation, behavioral influence, and cross-platform content patterns to better understand the impact of short-form health videos.

## 1. Introduction

Oil pulling is an ancient Ayurvedic technique in which individuals swill edible oil (often coconut or sesame) in their mouths for an extended period to purportedly remove toxins, reduce plaque, and improve overall oral health [1,2,3]. While historically practiced in parts of Asia, oil pulling has gained global visibility over the last decade, spurred partly by social media posts and anecdotal endorsements [4,5]. In particular, short-form video platforms have recently become influential spaces where oral health trends can achieve rapid, viral uptake [6,7].

YouTube Shorts, one of the most popular short video apps worldwide, enables users to create and share up to 60 s videos on nearly any topic, including alternative health practices. The ease of content creation and sharing has made YouTube Shorts a powerful vehicle for disseminating public health messages, but also for spreading misinformation if claims are not grounded in scientific evidence [8,9,10]. Several studies examining YouTube Shorts’s role in health information have identified both potential benefits, such as rapidly informing young audiences, and risks, which are oversimplified or misleading content, highlighting the platform’s growing relevance in shaping health behaviors [11].

The scientific literature on oil pulling is limited, consisting mainly of small-scale clinical studies that suggest potential antibacterial and anti-inflammatory effects but often lack robust sample sizes and rigorous methodologies. The peer-reviewed evidence on oil pulling remains sparse and methodologically heterogeneous. Early randomized, triple-blind trials by Asokan and colleagues, each enrolling only twenty adolescents, reported that daily sesame-oil pulling for 1–2 weeks reduced Streptococcus mutans counts in plaque and saliva by approximately 10^4^ CFU (*p* ≤ 0.01) and produced modest yet significant declines in plaque and modified gingival-index scores when compared with baseline, although chlorhexidine out-performed oil pulling at most time points [12]. A subsequent seven-day RCT in forty dental-student volunteers demonstrated that coconut-oil pulling lowered Quigley–Hein plaque scores by about 0.4 units versus placebo by day 7 (*p* < 0.001) [13]. In vitro work has shown that sesame oil itself lacks direct bactericidal activity; its apparent benefit may arise from an emulsification–saponification process that mechanically disrupts biofilm adherence rather than killing bacteria directly [14]. A 2022 meta-analysis pooling nine small RCTs (total *n* = 435) confirmed a reduction in salivary bacterial colony counts (mean difference ≈ 18 × 10^3^ CFU/mL) but detected no statistically significant improvement in plaque or gingival indices, underscoring both the short study durations (≤4 weeks) and the overall low certainty of the evidence [15]. Similarly, a 2016 narrative review concluded that while some studies reported improvements in plaque, gingival inflammation, and halitosis, the overall evidence remains inconsistent and insufficient to recommend oil pulling as a substitute for standard oral hygiene practices [3]. However, YouTube Shorts commonly portray oil pulling as a simple, low-cost intervention that may whiten teeth, freshen breath, or reduce gum inflammation, which are claims often made without professional dental guidance. As a result, those who encounter these contents on YouTube Shorts may adopt oil pulling in place of, or in addition to, conventional oral hygiene measures such as fluoride toothpaste, regular brushing, and professional cleanings [16,17,18].

Despite its uncertain evidence base, oil pulling trends continue to flourish on YouTube Shorts, where creative short videos can rapidly circulate among millions of users. This environment may facilitate unchecked propagation of anecdotal endorsements, further complicated by influencer-driven marketing [8,9,10]. Cross-sectional surveys can provide timely snapshots of these emerging behaviors, elucidating how often individuals encounter YouTube Shorts-based videos on oil pulling, what motivates them to try it, and how they perceive or experience changes in oral health [6].

YouTube Shorts has become a fertile environment that may facilitate unchecked propagation of anecdotal endorsements, further complicated by influencer-driven marketing. To understand how users may internalize and act upon these messages, Social Cognitive Theory (SCT) offers a useful framework. SCT posits that individuals learn behaviors through observing others, especially when the modeled behavior is perceived to be effective, socially approved, and attainable. These factors are often present in short-form videos that feature testimonial-style endorsements or repeated claims by influential creators. A recent SCT-based study found that perceived source credibility, positive outcome expectations, and self-efficacy significantly influenced health-related information-seeking and behavioral adoption via social media platforms, highlighting how persuasive social content can shape oral health behaviors, even in the absence of empirical support [19].

Accordingly, this study aims to analyze the content of YouTube Shorts videos on oil pulling to understand the prevalence of key practices promoted, assess the perceived benefits and drawbacks as presented in these videos, and examine how speaker type and stance influence the tone, credibility, and audience engagement of oil pulling content. By examining the role of YouTube Shorts in shaping public perceptions of oil pulling, this study seeks to inform dental professionals about the dynamics of online health messaging, support public health strategies for accurate information dissemination, and contribute to the evidence base surrounding alternative oral health practices.

## 2. Methods

**Figure 1:**
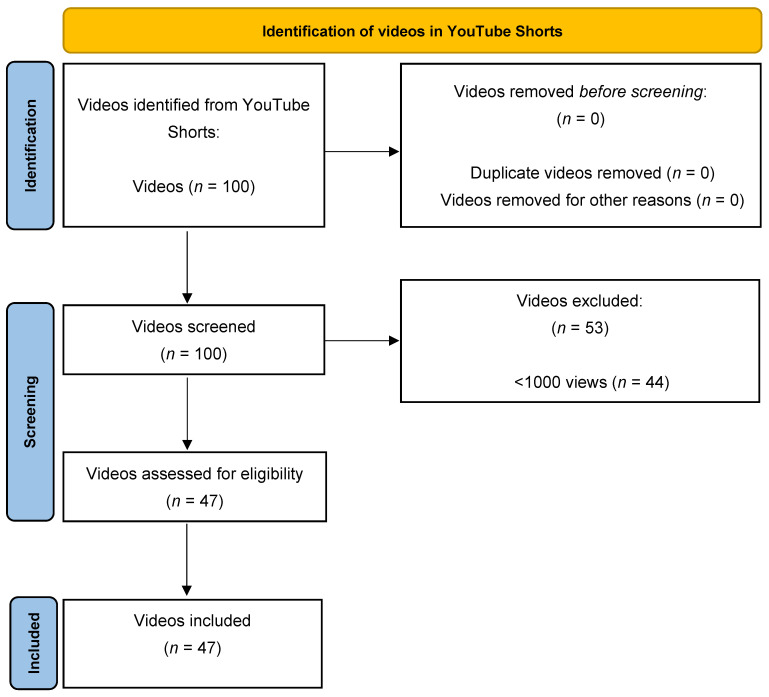
Flow diagram illustrating the PRISMA method systematic review for identification, screening, eligibility assessment, and inclusion of YouTube Shorts videos related to oil pulling.

This study employed the PRISMA method for a systematic analysis (meta and content analysis) to examine YouTube Shorts videos related to oil pulling. On 28 January 2025, a systematic search was performed using incognito mode to minimize algorithmic bias. The search term “oil pulling” was entered under the “Shorts” category on YouTube. The population for this study consisted of publicly accessible YouTube Shorts available globally at the time of the search. From the first 100 videos, videos were included if they were in English, publicly accessible, and had at least 1000 views to ensure relevance and visibility [20]. Exclusion criteria included non-English language, duplicates, or videos falling below the view threshold (Figure 1). Speaker nationality (e.g., US certified vs. non-US certified) was recorded only when publicly available on the speaker’s YouTube profile or linked website, rather than inferred. No geographic restriction was applied; however, speaker nationality or credential origin was documented when explicitly stated in the video content. Each eligible video was independently reviewed by two researchers using a structured coding sheet, and discrepancies were resolved through discussion and consensus.

The selection process followed a transparent, staged approach modeled on the PRISMA framework. First, all videos returned by the initial search on YouTube Shorts using the keyword “oil pulling” (*n* = 100) were identified as potentially relevant content. In the screening phase, titles, thumbnails, and brief descriptions were reviewed to remove clearly irrelevant or duplicate videos, resulting in 23 exclusions. During the eligibility assessment, the full content of the remaining 77 videos was evaluated against the predefined inclusion criteria, which required English language, public accessibility, and a minimum view threshold of 1000 views. Finally, 47 videos that met all criteria were included in the final analysis dataset.

Evaluator selection followed a purposive sampling approach. Both researchers had academic backgrounds in dental science and experience in health communication and content analysis, ensuring accurate interpretation of oral health claims and consistent classification of video features.

For each video included, data were extracted into a standardized spreadsheet using a structured coding framework. Recorded variables included speaker type (e.g., dentist, dental hygienist, influencer), engagement metrics (likes, comments, views), type and quantity of oil recommended, frequency and duration of oil pulling, stated benefits, and the presence of visual demonstrations. Videos were also evaluated for disclaimers or references to scientific literature. The speaker’s overall stance toward oil pulling was rated on a 5-point Likert scale (1 = “strongly discourages,” 5 = “strongly encourages”), adapted from established video content evaluation practices that assess video quality, flow, and perceived usefulness using similar ordinal measures [21,22].

Descriptive statistics, including frequencies and proportions, were used to summarize categorical data. Trends were analyzed by organizing findings according to speaker type, Likert score, and viewership. Engagement was calculated as [(likes + comments)/views] × 100 and reported as a percentage to provide insight into audience responsiveness [20].

Following data extraction, the dataset was systematically analyzed to identify content patterns and speaker messaging trends. Videos were grouped by speaker type and stance level, and the frequency of claims (e.g., purported benefits, drawbacks, presence of visual demonstrations) was tabulated. Comparative analyses were conducted to highlight variations between licensed professionals and non-professional influencers. In addition, recurring thematic narratives, such as the replacement of brushing or the promotion of exaggerated systemic benefits, were noted to contextualize broader messaging dynamics.

To ensure consistency in data categorization, a calibration exercise was conducted wherein a subset of videos was independently reviewed by both evaluators. Inter-rater reliability for the Likert ratings was assessed using Cohen’s kappa, which yielded a value of 0.83, indicating almost perfect agreement. Any discrepancies in labeling or classification were resolved through group discussion.

This study did not require review or approval by an institutional ethics review board because it involved the analysis of publicly available online content without interaction with human subjects or the collection of identifiable private data. All data were retrieved from YouTube Shorts videos accessible in the public domain, and user identities were anonymized during analysis in accordance with established ethical guidelines for social media research.

This methodology enabled a structured and transparent assessment of oil pulling narratives on YouTube Shorts, allowing for an evidence-based evaluation of content trends and potential public health implications. Given the exploratory and descriptive nature of this study and the relatively small sample size, inferential statistical analyses were not performed, as they were unlikely to yield stable or generalizable estimates.

## 3. Results

In reviewing 100 potential YouTube Shorts on oil pulling, researchers ultimately retained 47 that met our inclusion criteria (English language, ≥1000 views, and direct focus on oil pulling). The remaining 53 were excluded based on insufficient views, lack of English language, or irrelevance to oil pulling such as merely referencing “coconut oil” without any mention of oral health. Our final sample provided a cross-section of both professional and non-professional speaker types, as detailed in Table 1, and revealed considerable variability in the purported benefits, methods, and frequency recommendations for oil pulling.

### 3.1. Video Speaker Characteristics

A total of 47 YouTube Shorts on oil pulling were included. Table 1 summarizes the distribution of speaker types, as well as median engagement metrics. The most common speaker category was influencers (24/47, 50%), who typically had lower median likes (106) but a broad range of views (median 4882.5) compared to professionally affiliated speakers. US-certified dentists comprised the second-largest group (12/47, 25%), with a median of 625 likes and 34,169.5 views. Less frequent speaker categories (each representing 1 to 2 videos, ~2–4% of the sample) included Ayurvedic practitioners, dental hygienists, dental students, Doctor of Physical Therapy, and non-US certified practitioners. Median engagement scores (likes + comments/views) ranged from 1.33% (Doctor of Physical Therapy) to 5.85% (non-US certified naturopathic doctor), suggesting modest but variable viewer interaction.

### 3.2. Overall Stance Toward Oil Pulling

Table 2 displays the breakdown of Likert scores reflecting each video’s stance on oil pulling (1 = strongly discourage, 5 = strongly encourage). Videos created by US-certified dentists and dental hygienists generally expressed skepticism or mild discouragement, assigning oil pulling an average Likert score of approximately 2.3–2.5. In contrast, influencers, Ayurveda practitioners, and other holistic health advocates assigned higher scores (approximately 4.0), indicating stronger encouragement of the practice. Specifically, influencers (average Likert score 3.96) represented the largest group advocating strongly encouraging stances (Likert scores 4 or 5). Moreover, videos strongly encouraging oil pulling (Likert 5), predominantly uploaded by influencers or holistic practitioners, typically achieved higher median engagement (5.85%) compared to videos from dental professionals strongly discouraging the practice (Likert 1), which had a lower median engagement (1.42%). This difference underscores viewers’ potential attraction to positive, anecdotal content despite professional reservations.

### 3.3. Types of Oil and Recommended Practices

As shown in Table 3, 100% pure coconut oil was recommended most frequently (31/47, 66%), followed by sesame oil (7/47, 15%). Six videos (13%) mentioned alternative oils or oil blends (e.g., mustard oil, clove oil, or peppermint-infused blends). Three videos (6%) did not specify or recommend any particular oil.

Typical instructions across all videos involved “swishing” the oil in the mouth for 1–20 min daily or on most days of the week.

### 3.4. Perceived Benefits of Oil Pulling

Among the 47 analyzed videos, Table 4 highlights the top 9 reported benefits. The most frequently cited advantages included antimicrobial properties (17/47, 36.2%), with speakers commonly referencing coconut oil’s lauric acid or its purported ability to reduce *S. mutans*. Improved gum health (13/47, 27.7%) and reduced halitosis (16/47, 34.0%) were also frequently mentioned. Teeth whitening was cited by 9 videos (19.1%), whereas reduced plaque (7/47, 14.9%) and anti-inflammatory effects (7/47, 14.9%) appeared in fewer but still notable proportions. Additional claims included “overall oral hygiene improvement” (6.4%) and miscellaneous systemic benefits such as “weight loss”, “improved gut health”, and “boosted immunity” (12.8%). Influencers were predominantly responsible for promoting these broader claims, accounting for 15 out of 23 videos making strong or systemic claims (e.g., antimicrobial effects, cavity reversal, sinus clearing, remineralization). Companies and holistic practitioners occasionally supported these claims (2 videos each), whereas US-certified dentists and dental hygienists rarely endorsed these broader health benefits, typically exhibiting more conservative and evidence-based approaches.

### 3.5. Recommended Frequency, Duration, Quantity, and Use of Visual Demonstration

Of the 47 included videos, 27 (57.4%) explicitly mentioned a recommended duration for oil pulling. Reported times ranged from 1 to 20 min, with a mean duration of 13.9 min. Nine videos (19.1%) specified a frequency, most commonly phrased as “every morning” or “every day,” which in consensus equated to once per day. Additionally, 19 videos (40.4%) specified the quantity of oil used. Among these, 15 videos recommended one tablespoon (1 tbsp ≈ 15 mL), while two videos each recommended either one teaspoon (1 tsp ≈ 5 mL) or two tablespoons (2 tbsp ≈ 30 mL). Lastly, only three videos (6.4%) included any form of visual demonstration of results, such as “before-and-after” comparisons or video evidence of whitening effects. The majority of videos provided claims and instructions through narration or captions without visual proof of efficacy (Table 5).

### 3.6. Reasons Cited Against Oil Pulling

Although relatively few videos explicitly discouraged oil pulling, Table 6 shows that criticisms primarily originated from US-certified dentists and dental hygienists. The most common professional cautions were that oil pulling is “not a substitute for brushing or flossing” (8.5%), followed by concerns about “lack of strong scientific evidence” (6.4%). Additionally, dentists explicitly noted that oil pulling is “not a substitute for professional dental treatments” (4.3%) and cautioned viewers about potential unrealistic expectations or lack of demonstrated effectiveness (4.3%). This highlights dental professionals’ consistent emphasis on scientific credibility and caution compared to influencers’ more anecdotal endorsements.

## 4. Discussion

A total of 47 YouTube Shorts highlighting oil pulling were analyzed, revealing a predominantly encouraging stance toward the practice. As shown in Table 5, coconut oil was cited most frequently for its purported antimicrobial and cleansing properties, with many speakers suggesting a daily regimen of 10 to 15 min of swishing. Several videos mentioned benefits such as reduced halitosis and improved gum health, while others extended claims into teeth whitening, anti-inflammatory effects, and broader systemic advantages.

Despite the widespread positivity, a subset of videos voiced skepticism or discouragement, highlighting concerns about substituting oil pulling for professional dental care or lacking substantive scientific evidence. The environment of YouTube Shorts, characterized by short, engaging, and algorithmically promoted content, mirrors behavioral pathways described by SCT. When users observe influencers or semi-professional speakers presenting oil pulling in an appealing and testimonial-driven manner, they may adopt these behaviors based on perceived benefits, social proof, and frequency of exposure. This aligns with findings from recent SCT-based studies, which show that individuals are especially likely to adopt health behaviors modeled on social media when they perceive the source as credible, the outcomes as desirable, and the behavior as achievable [19]. Such modeling can amplify health behaviors even in the absence of rigorous evidence, reinforcing the need for critical evaluation and professional engagement in the online health space.

Interestingly, many of the Shorts uploaded by licensed dentists or dental hygienists tended to exhibit lower Likert scores of 2, indicating only mild or no support for oil pulling. Specifically, among US-certified dentists, antimicrobial properties and improved gum health were cited in only 2 videos each, whereas influencers mentioned antimicrobial properties (10 videos), reduced halitosis (13 videos), and improved gum health (7 videos) significantly more often. By contrast, influencers and other types of practitioners, including holistic or alternative health professionals such as Ayurvedic practitioners, naturopathic doctors, and doctors of physical therapy, generally favored oil pulling more strongly, frequently assigning it Likert scores of 4 or 5. This discrepancy suggests that formal dental training may predispose dentists and hygienists to be more cautious in their endorsements, likely reflecting the incomplete or inconclusive evidence supporting oil pulling as a standalone oral health intervention. In contrast, influencers may be more inclined to promote anecdotal and holistic claims, emphasizing personal experiences or perceived benefits. The observed differences in stance toward oil pulling between professional dental providers (average Likert ~2.3–2.5) and non-professional or holistic practitioners (average Likert ~4.0) illustrate the divergence between evidence-based dentistry and anecdotal or influencer-driven content. Influencers frequently advanced more extreme or systemic claims (e.g., cavity reversal, weight loss, improved gut health), whereas dental professionals were markedly more cautious in their endorsements, focusing primarily on scientifically supported oral hygiene practices. Notably, videos from influencers tended to receive higher engagement metrics (e.g., likes and comments relative to views) compared to those from dental professionals, although this difference was descriptive rather than statistically tested. These findings highlight the substantial impact of anecdotal content on public perceptions and behaviors. Accordingly, greater professional engagement on YouTube Shorts is needed to counterbalance misinformation and promote evidence-based oral health practices. From the perspective of this study, this divergence highlights the challenges consumers face when YouTube Shorts content offers mixed messages, one derived from evidence-based practice, another from personal or holistic narratives.

An important observation from our analysis was the clear disparity in viewership patterns between videos created by professionals versus influencers, as well as differences related to the speakers’ stance on oil pulling. Videos created by influencers, who generally took a strongly encouraging stance toward oil pulling (Likert scores 4–5), tended to receive moderate median views but collectively represented a substantial proportion of the total content. In contrast, videos featuring US-certified dental professionals and dental hygienists, who mostly expressed skepticism or mild discouragement (Likert scores 1–2), typically garnered higher median viewership per video. This difference may indicate that viewers attribute higher perceived credibility or authority to professionally credentialed speakers [23,24]. Alternatively, it could reflect the algorithmic amplification of professionally labeled content on platforms like YouTube Shorts. These findings underscore the importance of engaging credentialed health professionals in YouTube Shorts, as their content appears to achieve greater individual visibility, potentially offering a counterbalance to anecdotal or exaggerated claims commonly promoted by influencers.

One striking claim from a US board-certified dentist and German dentist in certain videos was that oil pulling can “reverse bad cavities” or “remineralize enamel”. While limited remineralization can occur under specific conditions such as adequate fluoride exposure and a favorable oral environment, these Shorts did not cite scientific studies to support the notion that swishing oil alone could halt or reverse established decay.

Such assertions may mislead viewers into delaying or foregoing professional care, echoing concerns reflected in Table 4, which highlights that oil pulling should not be treated as a substitute for standard oral hygiene measures. Even more concerning were videos suggesting that brushing could be completely replaced by oil pulling, directly challenging long-established preventive protocols in dentistry.

Beyond conventional oral health claims, a subset of video speakers promoted broader systemic benefits, including weight loss, sinus clearance, reduced bloating, improved immunity, and the healing of cracked lips. While Table 3 shows that antimicrobial properties and reduced halitosis were the most frequently cited advantages, which align somewhat with anecdotal reports, these broader claims lack robust clinical support. Moreover, some video speakers promoted oil pulling as a means to improve gut flora or encourage fuller cheeks, which may be an overstatement of the practice’s benefits.

Despite these outliers, the data in Table 2 indicate that most videos adopted a generally encouraging stance (Likert 4), with only a small fraction strongly discouraging oil pulling. Coconut oil, identified in Table 5 as the most common type recommended, was routinely associated with lauric acid’s presumed antibacterial effects [25,26]. Table 1 reveals that videos commonly propose 10–15 min of daily swishing, though exact frequencies and durations vary considerably, reflecting the inconsistency of videos on short-form platforms. Such variability underscores how anecdotal methods and influencer testimonials dominate the space, often outpacing evidence-based guidelines.

Table 6 further highlights the lack of standardization in how oil pulling is recommended across YouTube Shorts. While 57.4% of videos specified a recommended duration, the suggested time ranged widely from 1 to 20 min, with an average of 13.9 min. Only 19.1% of videos explicitly mentioned frequency, with most aligning around once daily use, typically phrased as “every morning” or “every day”. Recommendations for the quantity of oil also varied, with one tablespoon (≈ 15 mL) being the most common, though some videos advocated for as little as one teaspoon or as much as two tablespoons. Notably, only 6.4% of videos provided any visual demonstration of results, such as before-and-after comparisons or whitening evidence. Clinically, such variability may lead viewers to inappropriate expectations regarding the efficacy of oil pulling, potentially discouraging adherence to conventional, proven oral hygiene practices. This variability, paired with the limited use of demonstrable outcomes, reinforces the anecdotal and often unverified nature of claims made in this content, emphasizing the importance of critical evaluation by viewers and the need for clearer, evidence-based public messaging.

This study offers a snapshot of oil pulling presentations on YouTube Shorts but is limited by its cross-sectional design, which may not capture evolving trends or algorithmic changes. The use of incognito mode reduces, yet may not fully eliminate, personalized recommendations. Although a systematic content analysis was conducted, the sample was limited to 47 YouTube Shorts videos available at a single timepoint (28 January 2025), which may not reflect newly uploaded or removed content. The platform’s dynamic, algorithm-driven environment means video availability and visibility can change rapidly. Additionally, this study did not include direct respondents, such as viewers or content creators. As a result, it is not possible to assess how viewers interpret the information presented or whether it impacts their oral health behaviors. This absence introduces a potential bias, as the perceived influence and intent of content may differ from actual audience reception. Speaker nationality and professional background were inferred from publicly available profile descriptions or linked websites, which may not always be complete or accurate. While purposive sampling ensured relevance to the topic and minimum view count thresholds were applied to filter popular content, these methods do not ensure generalizability. Future research would benefit from incorporating mixed-method approaches, such as surveys or interviews with viewers and content creators, to better understand the motivations behind video creation, audience interpretation, and behavioral impact. This would complement the present study’s findings and offer a more holistic understanding of how oil pulling content is consumed and perceived on social media.

Nevertheless, these findings demonstrate the critical need for dental professionals to engage on YouTube Shorts to counteract misinformation. While oil pulling may provide adjunctive oral hygiene benefits, such as reducing halitosis and potentially decreasing the microbial load in the oral cavity [25,27], there remains little scientific consensus on its ability to reverse dental decay or thoroughly replace conventional oral hygiene practices like brushing with fluoride toothpaste or professional dental care [18,28]. Previous research indicates that exaggerated or misleading online content can lead individuals to delay seeking appropriate dental treatment, subsequently resulting in poorer oral health outcomes [29,30]. For instance, relying on oil pulling alone, in place of conventional practices, can result in inadequate plaque removal, thereby increasing the risk of dental caries and periodontal diseases [31]. A comparative clinical study in children demonstrated that while oil pulling with sesame oil reduced Streptococcus mutans levels, it was significantly less effective than fluoride mouthrinse in lowering caries activity, reinforcing that oil pulling should not be considered a substitute for evidence-based oral hygiene measures [32].

Misinformation propagated through YouTube Shorts may have tangible clinical consequences. When individuals replace conventional oral hygiene practices, such as brushing with fluoride toothpaste and routine professional care, with unverified alternatives like oil pulling, they may experience inadequate plaque control, increasing their risk for dental caries and periodontal disease. Indeed, poor oral hygiene has been shown to increase the risk of periodontitis by 2–5 times compared to good hygiene practices [33]. These behavioral shifts, encouraged by algorithmically favored, testimonial-heavy content, can delay early detection and management of oral diseases, thereby increasing long-term treatment burden. Studies on TikTok and Instagram have reported similar patterns of oral health misinformation, where unverified dental hacks or aesthetic “trends” receive disproportionate engagement compared to professional advice [34]. Like YouTube Shorts, these platforms promote content optimized for virality, not accuracy, compounding the challenge for public health communication [35]. Such cross-platform trends highlight the urgent need for digital health literacy education and more robust involvement from dental professionals across all major social media ecosystems.

## 5. Conclusions

In summary, while many YouTube Shorts endorse oil pulling as a beneficial oral care technique, only four videos—featuring one Ayurvedic influencer, two dental hygienists, and one non-dental influencer—explicitly claimed that scientific evidence supported its efficacy. However, none of these videos cited formal scientific references to substantiate their statements, instead offering verbal disclaimers such as “there is no scientific proof.” Moreover, some extended claims to reversing the formation of cavities, enhancing immunity, or eliminating the need for brushing. These inconsistencies highlight a broader issue of anecdotal health advice proliferating across short-form platforms. As interest in oil pulling grows, further research and professional engagement are needed to ensure potential benefits are neither overstated nor misconstrued at the expense of reliable, evidence-based oral care.

Grounded in Social Cognitive Theory (SCT), this study explores how modeled behaviors on YouTube Shorts, particularly by perceived credible figures such as influencers, can shape viewer beliefs and oral hygiene behaviors. The strong endorsements from non-professional speakers, amplified by platform algorithms, may drive health behavior adoption regardless of clinical evidence. To counteract this, future efforts should leverage SCT-informed strategies: increasing the visibility of dental professionals on short-form media, improving digital health literacy, and promoting accurate, accessible oral health messaging. These interventions are essential to guide viewers toward evidence-based practices and reduce the risk of misinformation-driven decisions in personal oral care.

These findings also illustrate how YouTube Shorts can serve as a practical medium to shape users’ knowledge, attitudes, and practices related to oral hygiene. By combining visually engaging content, testimonials, and perceived social proof, the platform has the potential both to encourage evidence-based behaviors and to propagate unsubstantiated claims such as oil pulling as a replacement for brushing. Future health communication efforts should leverage this medium to improve public knowledge and attitudes toward scientifically supported oral hygiene methods while proactively addressing misinformation in an accessible format.

## Figures and Tables

**Table 1:** Data of YouTube Shorts Recommendations on Oil Pulling.

Video Speaker	% (*n*)	Median Likes	Median Comments	Median Views	Median Engagement Score	Median Likert Scale
Influencer	50% (24)	106	7	4882.50	2.15%	4
US certified dentist	25% (12)	625	47	34,169.50	2.04%	2
Product Company *	4.2% (2)	4962.5	173	326,566.5	1.98%	4
Dental hygienist	4.2% (2)	561	16.5	23,986.5	2.39%	2.5
Ayurveda practitioner	2% (1)	1500	17	45,591	3.33%	4
Dental student	2% (1)	13,000	231	286,371	4.62%	2
Doctor of Physical Therapy	2% (1)	26	2	2102	1.33%	4
Doctorate Degree in Naturopathy and Yogic Sciences/Certified NLP practitioner	2% (1)	73	5	4434	1.76%	4
Medical clinic	2% (1)	91	9	5752	1.74%	3
Non-US certified naturopathic doctor	2% (1)	618	16	10,843	5.85%	5
Non-US certified dentist, Germany	2% (1)	113	2	4006	2.87%	4

* Product companies included ScanO and Banyan Botanicals.

**Table 2:** Likert Scale Distribution of Stance on Oil Pulling.

Likert Scale	Interpretation	Number of Videos	Median Likes	Median Comments	Median Views	Median Engagement
1	Strongly discourage	3	250	65	28,084	1.42%
2	Somewhat discourage	8	1500	48.5	59,280	2.53%
3	Neutral/ No stance	4	224.5	17.5	11,178.50	2.01%
4	Somewhat encourage	25	102	6	4706	2.25%
5	Strongly encourage	5	391	13	3852	5.85%

**Table 3:** Type of Oil Recommended in YouTube Shorts.

Oil Type	Number of Videos	% of Videos
100% Pure Coconut Oil	31	66%
Sesame Oil	7	15%
Other ^†^	6	13%

^†^ Other oil pulling types include oil (not specified), olive oil, mustard oil, gingelly oil, and peppermint oil.

**Table 4:** Top Perceived Benefits of Oil Pulling Mentioned in YouTube Shorts.

Top 9 Perceived Benefits of Oil Pulling	Number of Videos Mentioning Specific Benefit (*n* = 47)	Percentage of Videos Mentioning Specific Benefit (*n* = 47)	Number of Video Speakers Recommending This Treatment								
			Influencer (*n* = 24)	US Certified Dentist (*n* = 12)	Product Company (*n* = 2)	Ayurveda Practitioner (*n* = 1)	Doctor of Physical Therapy (*n* = 1)	Doctorate Degree in Naturopathy and Yogic Sciences/Certified NLP Practitioner (*n* = 1)	Medical Clinic (*n* = 1)	Non-US Certified Naturopathic Doctor (*n* = 1)	Non-US Certified Dentist, Germany (*n* = 1)
Antimicrobial properties ‡	17	36.17%	10	2	2	1	1	1			
Reduce halitosis	16	34.04%	13		2			1			
Improve gum health (including reduces gum recession)	12	25.53%	7	2				1	1		1
Teeth Whitening	8	17.02%	6	1						1	
Reduce cavity	7	17.02%	5		1			1			
Reduce plaque	7	14.89%	4		1				1	1	
Anti-inflammatory	7	14.89%	4		1		1			1	
Miscellaneous ^§^	6	12.77%	4	1			1				
Overall oral hygiene improvement	3	6.38%	1	1					1		

‡ Antimicrobial properties include Lauric acid in coconut oil, reduction of *S. mutans*, detoxification. ^§^ Miscellaneous includes improvement of gut health or oral microbiome, weight loss, etc.

**Table 5:** Recommended Frequency, Duration, Quantity, and Use of Visual Demonstration in YouTube Shorts on Oil Pulling.

Parameter	Number of Videos (*n* = 47)	% of Videos	Details/Distribution
Frequency Mentioned	9	19.1%	“Every morning” or “Every day”
Duration Mentioned	27	57.4%	Range: 1–20 min Mean: 13.9 min
Quantity Mentioned	19	40.4%	2 videos: 1 tsp (≈5 mL) 15 videos: 1 tbsp (≈15 mL) 2 videos: 2 tbsp (≈30 mL)
Visual Demonstration of Results	3	6.4%	Videos of before-and-after comparison

**Table 6:** Reasons Cited Against Oil Pulling Among YouTube Shorts.

Reasons Cited Against Oil Pulling	Number of Videos Against Oil Pulling (*n* = 47)	Percentage of Videos Against Oil Pulling (*n* = 47)
Not a substitute for brushing/flossing	4	8.51%
No strong scientific evidence supporting the claims	3	6.38%
Not a substitute for dental treatments	2	4.26%
Results weren’t effective	2	4.26%

## Data Availability

The original data presented in the study are openly available in Figshare at https://doi.org/10.6084/m9.figshare.29605031, dataset posted on 20 July 2025.
